# Efficacy and safety of Tuina for chronic nonspecific low back pain: A PRISMA-compliant systematic review and meta-analysis

**DOI:** 10.1097/MD.0000000000033018

**Published:** 2023-03-03

**Authors:** Juan Yang, Xuan Zhou, Qingyu Ma, Jeffrey T. Woods, Arya B. Mohabbat, Alexander Do, Jeffrey S. Brault, Mark A. Jensen, Kyung-Min Shin, Longbin Shen, Canghuan Zhao, Kwok Chee Philip Cheong, Kejie He, Yu Guo, Zhuoming Chen, Shujie Tang, Yong Tang, Celia Ia Choo Tan, Jiaxu Chen, Brent A. Bauer

**Affiliations:** a Division of General Internal Medicine, Mayo Clinic, Rochester, MN; b Formula-pattern Research Center, School of Traditional Chinese Medicine, Jinan University, Guangzhou, China; c Mayo Clinic Alix School of Medicine, Rochester, MN; d Department of Physical Medicine and Rehabilitation, Mayo Clinic, Rochester, MN; e KM Science Research Division, Korea Institute of Oriental Medicine, Daejeon, South Korea; f Department of Rehabilitation Medicine, The First Affiliated Hospital of Jinan University, Guangzhou, China; g Department of Acupuncture, The First Affiliated Hospital of Jinan University, Guangzhou, China; h Department of Physiotherapy, Singapore General Hospital, Singapore; i Department of Orthopedics, School of Traditional Chinese Medicine, Jinan University, Guangzhou, China.

**Keywords:** Chinese massage, chronic nonspecific low back pain, low back pain, review, Tuina

## Abstract

Chronic nonspecific low back pain (CNLBP) is a serious medical and social problem resulting in functional decline and decreased work ability. Tuina, a form of manual therapy, has been sparsely used to treat patients with CNLBP. To systematically assess the efficacy and safety of Tuina for patients with CNLBP.

**Methods:**

Multiple English and Chinese literature databases were searched until September 2022 for randomized controlled trials (RCTs) of Tuina in the treatment of CNLBP. The methodological quality was assessed using the Cochrane Collaboration’s tool, and certainty of the evidence was determined with the online Grading of Recommendations, Assessment, Development and Evaluation tool.

**Results:**

Fifteen RCTs with 1390 patients were included. Tuina demonstrated a significant effect on pain (SMD: −0.82; 95% CI −1.12 to −0.53; *P* < .001; *I*^2^ = 81%) and physical function (SMD: −0.91; 95% CI −1.55 to −0.27; *P* = .005; *I*^2^ = 90%) when compared to control. However, Tuina resulted in no significant improvement for quality of life (QoL) (SMD: 0.58; 95% CI −0.04 to 1.21; *P* = .07; *I*^2^ = 73%;) compared to control. The Grading of Recommendations, Assessment, Development and Evaluation evidence quality was determined to be low level for pain relief, physical function, and QoL measurements. Only six studies reported adverse events; none were serious.

**Conclusion:**

Tuina might be an effective and safe strategy for treating CNLBP in terms of pain and physical function, but not for QoL. The study results should be interpreted with caution for their low-level evidence. More multicenter, large-scale RCTs with a rigorous design are required to further confirm our findings.

## 1. Introduction

Low back pain (LBP) refers to pain and discomfort that is localized below the costal margin and above the inferior gluteal folds, with or without referred leg pain.^[1]^ As one of the most common reasons for seeking medical care worldwide, LBP is the leading cause of disability around the world, affecting about 540 million people.^[2]^ Approximately 90% to 95% of LBP is not attributed to a specified pathological factor and is termed nonspecific low back pain.^[3]^ If the nonspecific low back pain persists for 12 weeks, it is defined as chronic nonspecific low back pain (CNLBP).^[4]^ CNLBP includes discogenic pain, lumbar muscle strain, zygapophyseal joint pain, and sacroiliac joint pain. It is influenced by complex central and peripheral nociceptive processes, as well as psychosocial and musculoskeletal factors. CNLBP often causes pain and functional disability, which has a detrimental impact on an individual’s quality of life (QoL).

The management of CNLBP is challenging for patients, researchers, and clinicians. Nonsteroidal anti-inflammatory drugs, antidepressants, psychosocial interventions, exercise therapy as well as complementary and alternative medicine (CAM) options are the most common interventions recommended to patients with CNLBP.^[4,5]^ However, little is known about which intervention is more effective than others.^[6]^

As one of the CAM therapies, Tuina has been used widely in China for over 5000 years. It is a manual therapy that features a series of manipulations of the body, including pressing, rubbing, pulling, and pinching of soft tissues and may also incorporate rotation or manipulation of extremities. The goal of these maneuvers is to reduce pain while also having a direct impact on the underlying disease process. Tuina has been shown to be effective for many clinical conditions, such as cervical vertigo,^[7]^ hypertension,^[8]^ chronic fatigue syndrome,^[9]^ chronic neck pain,^[10]^ peripheral nerve injury,^[11,12]^ headache,^[13]^ and various musculoskeletal conditions.^[14]^ It has also become an important treatment method for LBP.^[15]^ Preliminary studies indicate the effectiveness of Tuina, as a stand-alone therapy or in combination, in patients with LBP.^[16,17]^ Tuina has been widely recommended to patients with LBP throughout Asian countries. Several studies previously reported the therapeutic effect of Tuina in patients with CNLBP.^[18–23]^ However, Therapeutic evaluation of lumbar tender point deep massage for chronic nonspecific low back pain most of these trials utilized Tuina in combination with other CAM therapies^[22,23]^ or were conducted with small sample sizes with poor methodological quality.^[18,19]^ Despite frequent reports of Tuina’s potential utility in CNLBP, there is a need for a comprehensive systematic review of the currently available evidence of Tuina in CNLBP. Therefore, this systematic review was conducted to evaluate the safety and efficacy of Tuina in CNLBP and to present up-to-date evidence that could be helpful for both clinical practice and future investigations.

## 2. Methods

### 2.1. Study protocol

This study complied with our registered protocol in the International Prospective Register of Systematic Reviews database (https://www.crd.york.ac.uk/prospero/display_record.php?ID=CRD42020166731). This study adhered to the Preferred Reporting Items for Systematic Reviews and Meta-Analyses statement.^[24]^

### 2.2. Data sources and searches

Multiple electronic database searches were conducted from database inception to September 2022. Databases included Embase, EBM Reviews – Cochrane Central Register of Controlled Trials, Cochrane Database of Systematic Reviews, Ovid MEDLINE(R) and Epub Ahead of Print, In-Process, In-Data-Review and Other Non-Indexed Citations and Daily. In addition, we also searched four Chinese databases (Wan-fang Database, Chinese Scientific Journals Database, China Biomedical Literature Database, and China National Knowledge Infrastructure). Manual searches for relevant clinical trials from the reference lists of previously published studies were also performed. Key terms related to Tuina, massage, low back pain, and lower back pain were included. The search was designed and performed by a Mayo Clinic librarian with input from the principal investigator. The search strategy used in OVID is available in the appendix.

### 2.3. Inclusion and exclusion criteria

Studies meeting the following inclusion criteria were included in this review: randomized controlled trials (RCTs) with Tuina as the primary treatment intervention for CNLBP; participants aged 18 years or older with a diagnosis of CNLBP; interventions including any type of Tuina or Tuina combined with other therapies; studies with a control/comparison group; and studies with primary outcomes of pain, physical function, and QoL. Other outcomes, such as adverse events (AEs) associated with Tuina, were considered as secondary outcomes. Exclusion criteria consisted of: studies involving participants without CNLBP; studies that included Tuina in the control group; studies that compared different Tuina techniques; non-RCTs or quasi-RCTs; published languages other than English nor Chinese; and animal or cellular level studies.

### 2.4. Data extraction and synthesis

Two reviewers independently screened the initially identified studies based on their titles and abstracts according to the inclusion and exclusion criteria. Full-text articles of included studies were further checked for potential inclusion. Any disagreement or discrepancy between the two reviewers was resolved by consensus or by the decision of a third reviewer. Quantitative data was statistically. The pain intensity, physical function, and QoL outcomes were recorded as continuous variables. Mean difference before and after treatment was used to pool differences between the experimental and control groups for each study. Outcome data were expressed with the standardized mean difference (SMD) and 95% confidence interval (CI) to standardize the study results into a uniform scale. Standard deviations were used for analyses and standard errors were converted to standard deviations when they were the only values presented. The I^2^ test was used to test statistical heterogeneity. In cases of high statistical heterogeneity, subgroup analyses on intervention type using the random effect model were performed. Sensitivity analysis was conducted by excluding each study individually to examine the stability of our findings. Statistical significance was defined with alpha threshold at 0.05. Publication bias was conducted with funnel plots if there were at least ten studies. Statistical analysis was conducted using R software (version 4.1.0). Meta-analysis was performed using the R package “meta.” (v4.18-1; Balduzzi et al, 2019).^[25]^ For studies with insufficient information, the original correspondence authors were contacted for data verification. Sensitivity analysis was carried out for primary outcome by removing each study individually. Subgroup analyses were performed to detect the sources of heterogeneity. Publication bias was also assessed by determining if the data points formed a symmetric funnel shaped. Publication bias was evaluated with funnel plots for the study outcomes, when at least ten trials were included.

### 2.5. Methodological quality assessment and the level of evidence

Risks of bias of each study was evaluated with the Cochrane Collaboration’s tool.^[26]^ Each of the six domains was rated as high risk, unclear risk, or low risk. Results were entered into the Review Manager (RevMan) Version 5.4.1, The Cochrane Collaboration, 2020.^[27]^ Study evidence quality for outcomes was assessed with the online Grading of Recommendations, Assessment, Development and Evaluation (GRADE) tool.^[28]^ The study outcome evidence was rated as very low, low, moderate, and high certainty. Any discrepancy between the two reviewers was resolved through a consensus discussion or by involving a third reviewer.

## 3. Results

### 3.1. Study selection and screening process

**Figure 1.**
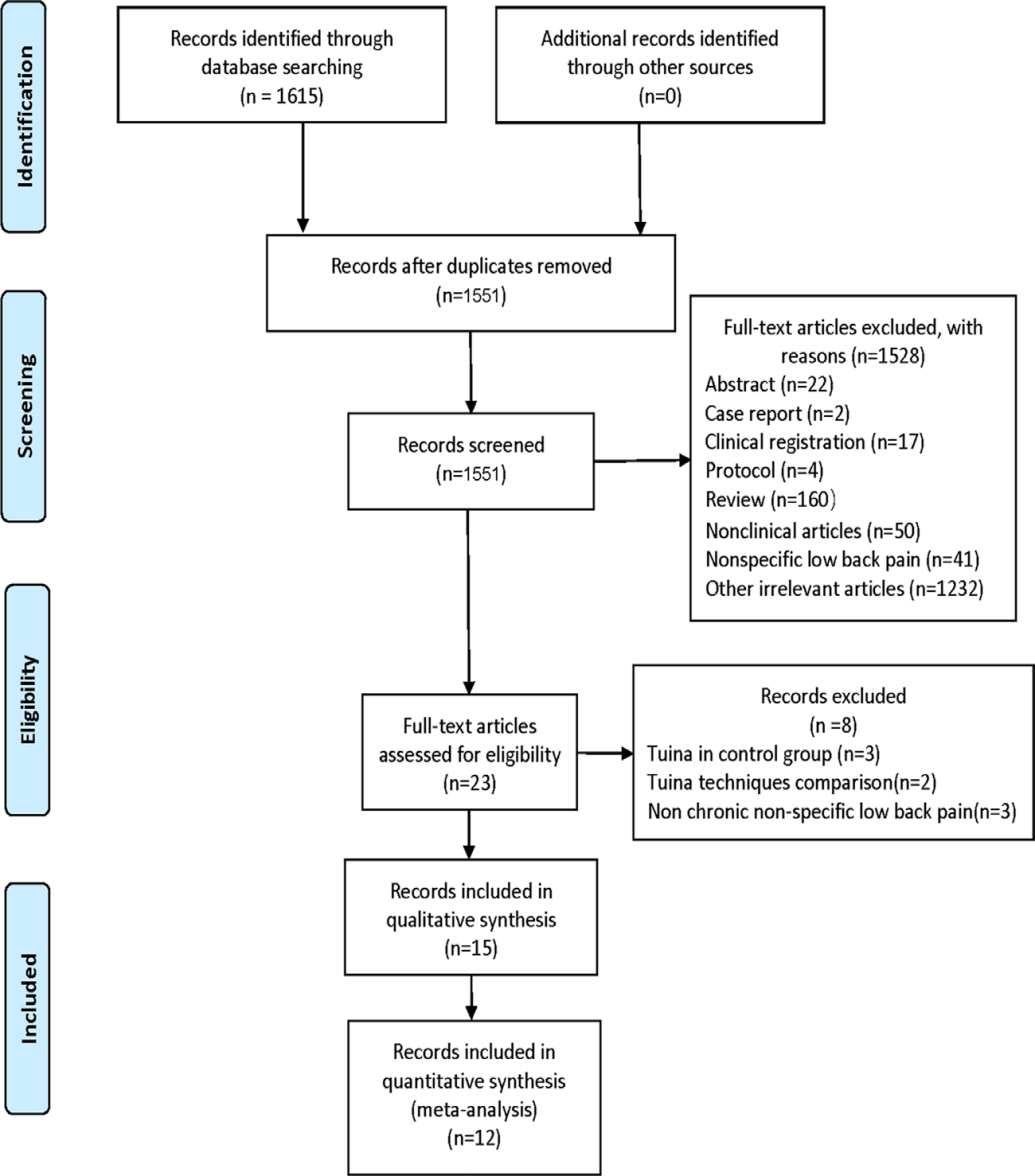
Flow chart of included studies.

According to the above search strategy, a total of 1615 records from the databases were obtained. Among them, 64 records were removed because they were identified as duplicate records. In total, 1528 records were excluded after screening the abstracts and titles by two reviewers, based on the inclusion and exclusion criteria. Of the remaining 23 articles, 8 were excluded from the study due to trials not involving participants with CNLBP, trials with Tuina in the control group, and trials comparing different Tuina techniques. Overall, 15 trials meeting the eligibility criteria were included in this systematic review, while 12 of these trials were included in the meta-analysis (Fig. 1).

### 3.2. Study characteristics

A total of 15 RCTs, consisting of 1390 patients were included in this review. All studies were published from 2005 to 2020. The sample size of each trial ranged from 60 to 128. All participants were adults aged from 18 to 78 years old. The included study population demonstrated similarities regarding the diagnosis of CNLBP but differed with respect to pain duration and severity. Among the included trials, 3 of them were three-armed studies,^[29–31]^ and the other 12 were two-armed trials.^[18,19,21,32–40]^ Six trials involved Tuina alone interventions,^[18,29,32,34,35,38]^ while the other 9 studies studied the effect of Tuina in combination with other treatments.^[19,21,30,31,33,36,37,39,40]^ For the outcomes, all studies assessed pain intensity and physical function over time, while 8 trials reported subjective symptom improvement rates.^[19,29–31,36,37,40]^ Two trials evaluated general health and QoL via the 36-Item Short Form Survey.^[36,39]^ Three trials reported inflammatory markers^[39]^ and electromyographic changes.^[35,40]^ Five trials reported follow-up varying from 1 month to 22 months,^[18,21,30,32,39]^ while the remaining did not record any follow-up data. AEs were reported in 6 trials,^[19,31–34,39]^ while 2 trials mentioned no AEs.^[31,34]^ Nine trials did not include any AE information.^[18,21,29,30,35–38]^ Treatment group duration varied amongst studies, ranging from 10 days to 3 months. All trials were published in Chinese except 2 studies,^[19,40]^ which were published in English. The detailed study characteristics are summarized in **Tables 1 and 2**.

**Table 1:** Characteristics of the included studies.

Author/year	Arm	N	Age	Groups	Outcome measurement
Han 2005^[18]^	2	122	22–49	Tuina (N = 60/62): 25–30 min/session, 10 sessions/treatment course for 1–3 courses High voltage medial frequency treatment (N = 60/60): 25–30 min/session, 10 sessions/treatment course for 1–3 courses	The Low-Back Outcome Score
Zheng 2012^[19]^	2	64	21–72	Lumbar tender point deep tissue massage plus lumbar traction (N = 30/32): 8–10 s/time for 4–5 times plus 20 min lumbar traction once daily, twice per week for 3 wk Lumbar traction (N = 30/32): 20 min lumbar traction once daily, twice per week for 3 weeks	VAS, The tissue hardness meter/algometer
Yuan 2014^[32]^	2	110	28–60	Tuina (N = 53/55): 30 min/session, 3 sessions/wk for 4 wk Health education (N = 51/55): 30 min/wk for 4 wk	VAS, ODI
Sun 2015^[21]^	2	120	33–47	Sling exercise therapy and Tuina (N = 44/60): Twice weekly for 4 wk Celebrex capsule (N = 48/60): 200 mg/d for 4 wk	VAS, ODI
Feng 2016^[29]^	3	90	36–59	Sling and Plucking Corresponding Meridian (N = 29/30): 30–60 min, once daily, 5 sessions/wk/treatment course for 3 courses Plucking therapy corresponding meridian (N = 26/30): 30–60 min, once daily, 5 sessions/wk/treatment course for 3 courses Sling exercise therapy group (N = 28/30): 30–60 min, once daily, 5 sessions/wk/treatment course for 3 courses	VAS, The JOA Back Pain Evaluation Questionnaire, Back extensor muscle strength and the flexibility of lower limb
Wang 2017^[30]^	3	96	42–48	Shen’s Mang-needle combined Tuina on belt vessel (N = 32/32): Shen’s Mang-needle 24 min/session daily for 2 wk + Tuina on belt vessel: 20 frequency/min, 5 times once, twice daily for 2 wk Tuina on belt vessel (N = 32/32): 20 frequency/min, 5 times once, twice daily for 2 wk Shen’s Mang-needle (N = 32/32): 24 min/session, once daily for 2 wk	VAS, Efficacy criteria
Chen 2018^[33]^	2	120	23–57	Sinewing tendon therapy plus cupping on Bladder meridian (N = 60/60): 30 min once/4 d for 3 mo Zhuanggu Guanjie Wan (N = 60/60): 5 g once, three times per day for 3 mo	VAS, Guidelines for Clinical Research on Soft Tissue Injury of New Chinese Medicine
Jia 2018^[34]^	2	68	39–63	Amelioration therapy of Gongting Therapeutic for Injured Soft Tissue (N = 30/36): 6 times within 4 wk Acupuncture therapy (N = 30/32): 20 min acupuncture with BPM Infra-Red Spectrometer for 6 times within 4 wk	The NRS scores, ODI index and the scores of self-rating disability scale
Liu 2018^[35]^	2	60	25–75	Dredging meridian and regulating viscera therapy (N = 30/30): Once daily, 10 sessions/treatment course for 2 courses Lumbago ning capsule (N = 30/30): 4 capsules once daily, 10 sessions/treatment course for 2 courses	VAS, Traditional Chinese Medicine syndrome score and Electromyography
Yu 2019^[31]^	3	81	20–48	Exercise intervention + acupuncture and massage (N = 27/27): Acupuncture + Tuina 30 min, then exercise 30 min, in total 60 min/session, 3 sessions/wk for 4 wk Exercise (N = 27/27): 30 min/session, 3 sessions/wk for 4 wk Acupuncture and Tuina (N = 27/27): 30 min (Tuina 15 min + acupuncture 15 min), 3 times/wk for 4 wk	VAS, ODI, core muscle endurance and explosive strength test, therapeutic effect, and recurrence rate
Jiang 2019^[36]^	2	128	25–70	Dredging meridian and regulating viscera therapy + Cheezheng pain relieving plaster (N = 64/64): once daily, 10 d/treatment course for 2 courses Cheezheng pain relieving plaster (N = 64/64): 1 plaster/24 h, 10 d/treatment course for 2 courses	VAS, SF-36, Clinical efficacy
Zhang 2019^[37]^	2	86	44–78	Musk pain relief plaster + Dredging meridian and regulating viscera therapy (N = 43/43): 1 plaster + 20 min Tuina daily for 20 d Musk pain relief plaster (N = 43/43): 1 plaster daily for 20 d	VAS, ODI, Traditional Chinese medicine syndrome score, total therapeutic efficacy rate
Lin 2020^[38]^	2	120	28–65	Therapeutic manipulation for tendon injury of Tuina (N = 60/60): 30 min once daily for 1 mo Routine rehabilitation training (N = 60/60): 30 min once daily for 1 mo	Self-made chronic nonspecific low back pain symptoms and signs observation scale; Japanese Orthopaedic Association Scores
Feng 2020^[39]^	2	60	18–58	Channels-acupoints dredge plus pain point knead dial Tuina manipulation combined with core stability training (N = 29/30): 30 min once daily for 2 wk Core stability training (N = 28/30): 30 min once daily for 2 wk	VAS, Roland-Morris Disability Questionnaire, SF-36, β-EP, IL-6, TNF-α
Yao 2020^[40]^	2	65	24–40	Sinew-regulating bone-setting manipulations plus exercise therapy (N = 31/33): Tuina 10–15 min with moderate force, once every other day with 7 sessions/treatment course; Exercise 10 times as 1 set, 3 sets daily, and 7 sessions/treatment course Medium-frequency electrotherapy plus exercise therapy (N = 30/32): 20 min/session once daily, 14 sessions as 1 treatment course. Exercise: 10 times as 1 set, 3 sets daily, and 7 sessions/treatment course	VAS, ODI, dynamic and static muscle endurance of low back, median frequency of surface electromyography

AE = adverse event, CNLBP = chronic nonspecific low back pain, IL-6 = interleukin-6, JOA = Japanese Orthopaedic Association Scores, NRS = Numeric Rating Scale, ODI = Oswestry Disability Index, QoL = quality of life, SF-36 = 36-Item Short Form Survey, TNF-α = tumor necrosis factor alpha, VAS = Visual Analog Scale, β-EP = β-endorphin.

**Table 2:** Characteristics of the study interventions.

Author/year	Follow-up	Compliance	Adverse events	Conclusions	Quality assessment
Han 2005^[18]^	Average period 22 mo	100%	/	Tuina had a good effect on CNLBP	Poor
Zheng 2012^[19]^	No detail for follow up time	TG: Discontinued intervention (2) CG: Lost to follow-up (2)	Low back pain got worse after deep massage, so stopped treatment in Tuina group (n = 2).	Lumbar tender point deep massage plus lumbar traction was superior to lumbar traction alone in pressure pain threshold, muscle hardness and pain intensity CNLBP patients	Poor
Yuan 2014^[32]^	1 mo	TG: Discontinued intervention (2), lost to follow-up (3) CG: Discontinued intervention (4), lost to follow-up (2)	Lumbar skin pain due to local manipulation the next day after Tuina treatment, it disappeared the next day without treatment.	Tuina was more effective than health education can in low back pain and activity in chronic lumbar muscle strain patients.	Poor
Sun 2015^[21]^	12 wk	TG: Discontinued intervention or lost to follow-up (16) CG: Discontinued intervention or lost to follow-up (12)	/	Sling exercise therapy plus Tuina was superior to conventional medication in symptoms improvement among CNLBP patients.	Poor
Feng 2016^[29]^	1 mo	TG: Discontinued intervention or lost to follow-up (1) CG: Discontinued intervention or lost to follow-up (6)	/	Sling and plucking corresponding meridian effectively eased the pain and improved back extensor muscle strength and functional state in patients with chronic lumbar strain.	Poor
Wang 2017^[30]^	/	100%	/	Shen’s Mang-needle combined Tuina on belt vessel was more remarkable and reliable effective in treatment of CNLBP patients.	Poor
Chen 2018^[33]^	/	100%	Purplish skin after Sinewing tendon therapy plus moving cupping on Bladder meridian, 2 patients reported lumbar discomfort.	Sinewing tendon therapy plus moving cupping on Bladder meridian could inhibit the release of inflammation factors IL-6, TNF-α and relieve pain of chronic lumbar strain patients.	Poor
Jia 2018^[34]^	No detail	TG: Discontinued intervention for personal reason (3), failure to complete within 4 weeks (3) CG: Discontinued intervention (2) for personal reason	No	Amelioration therapy of Gongting Therapeutic for Injured Soft Tissue was safe and effective for CNLBP and worthy of clinical promotion.	Poor
Liu 2018^[35]^	/	100%	/	Dredging meridian and regulating viscera therapy could relieve pain of chronic lumbar strain patients in smoothing their lumbar spasm and improving qi and blood circulation.	Poor
Yu 2019^[31]^	/	100%	No	The combination of exercise intervention and acupuncture and massage is more cost-effective for NLBP patients	Poor
Jiang 2019^[36]^	/	100%	/	Cheezheng pain relieving plaster plus dredging meridian and regulating viscera therapy could relieve lumber pain and QoL of patients with chronic lumbar strain.	Poor
Zhang 2019^[37]^	/	100%	/	Musk pain relief plaster plus Dredging meridian and regulating viscera therapy could relieve lumbar pain and improve lumbar activity among patients with chronic lumbar strain	Poor
Lin 2020^[38]^	/	100%	/	Therapeutic manipulation for tendon injury of Tuina could effectively enhance spinal function and recover the stability of spine of patients with CNLBP.	Poor
Feng 2020^[39]^	1 year	TG: Discontinued intervention due to business trip (1). CG: Discontinued intervention due to inability to complete the core stabilization exercise waist training (2)	OG: NO; CG: Lumbar pain and inability severed after core stability training, but relieved after 1-day flat bed rest	channels-acupoints dredge plus pain point knead dial massage manipulation combined with core stability exercise had a significant effect in lumbar pain, function, and QoL.	Poor
Yao 2020^[40]^	/	TG: Discontinued intervention with personal reasons (2) CG: Discontinued intervention (1), 1 patient added other treatments without approval (1)	/	Sinew-regulating bone-setting manipulations plus exercise therapy could effectively release pain in CNLBP patients, increase muscle endurance of the low back and improve the QoL	Poor

AE = adverse event, CG = control group, CNLBP = chronic nonspecific low back pain, TG = treatment group.

### 3.3. Quality assessment

**Figure 2.**
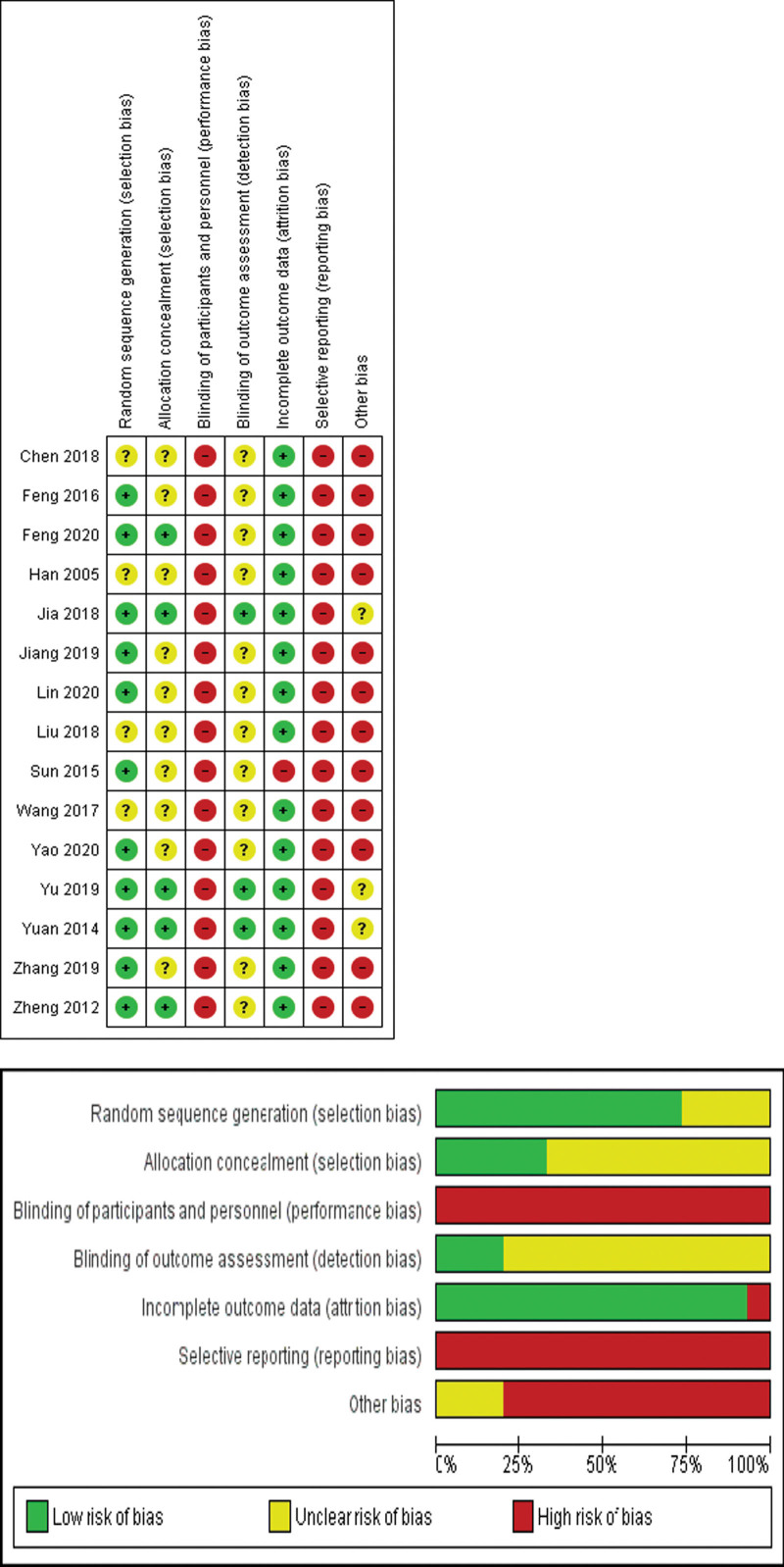
Risk of bias summary of included trials.

Four studies did not report details regarding random number generation; thus, these trials were deemed as “unclear risk” of bias.^[18,30,33,35]^ The other trials described random sequence generation in detail during the study,^[19,21,29,31,32,34,36–40]^ including a random number generator in 9 studies,^[21,29,31,34,36–40]^ random numbers generated using Microsoft Office Excel^[19]^ and SAS SoftWear^[32]^; thus these trials were judged as “low risk” of bias. Five studies recorded allocation concealment with opaque sealed envelopes^[31,32,34,39]^ or an allocation table,^[19]^ which were assessed as “low risk” in terms of selection bias. The other trials did not report the detailed information of the intervention and thus were rated as^[18,21,29,30,33,35–38,40]^ “high risk.” Three studies were assessed as “low risk” in terms of blinding of outcome assessment,^[31,32,34]^ while the other as “high risk” due to a lack of blinding information, leading to both performance bias and detection bias. In terms of attrition bias, one trial was identified as “high risk” for incomplete outcome data or incomplete outcome data not being addressed adequately,^[21]^ while the remaining studies were regarded as “low risk” given their complete outcome data.^[18,19,29–40]^ Due to a lack of protocol or registration information, all included trials were assessed as “high risk” in terms of reporting bias. Regarding the assessment of other bias, three of the included trials had an “unclear risk” of other bias,^[31,32,34]^ while the other trials had “high risk” of other bias.^[18,19,21,29,30,33,35–40]^ The overall assessment of risks of bias was rated as poor (Fig. 2).

### 3.4. Meta-analysis and quality of evidence

#### 3.4.1. The efficacy of Tuina on pain in patients with CNLBP.

All studies compared the potential pain reduction properties of Tuina to that of a control group. The Visual Analog Scale and Numeric Rating Scale were used to determine the efficacy of Tuina alone therapy or Tuina combined therapy for CNLBP in 13 trials. Only 12 studies were pooled in the meta-analysis,^[19,21,29,31–34,36,37,39,40]^ due to the remaining trial not reporting detailed baseline data.^[35]^ Four trials investigated the effect of Tuina alone therapy on pain reduction,^[29,30,32,34]^ while ten trials investigated the effect of Tuina combined therapy.^[19,21,29,30,32,33,36,37,39,40]^ Comparison groups consisted predominantly of multiple interventions (i.e., exercise, acupuncture, traction, herbal medication, health education, and other combined treatment) in CNLBP.

**Figure 3.**
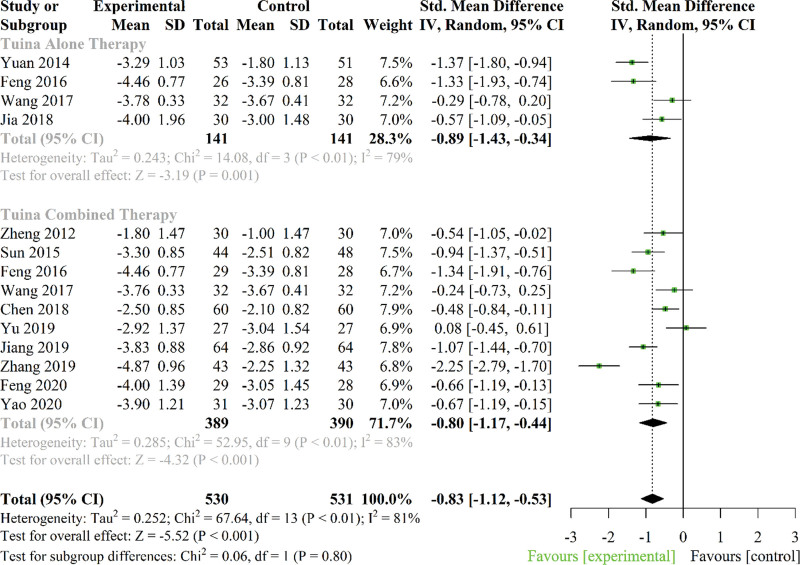
Forest plot of the effect of Tuina on pain in patients with chronic nonspecific low back pain. CI = confidence interval, SD = standard deviation.

Pooled analysis revealed Tuina therapy significantly decreased pain compared to control groups in CNLBP patients (SMD: −0.83; 95% CI −1.12 to −0.53; *P* < .001; *I*^2^ = 81%). Subgroup analysis was performed based on Tuina alone therapy or Tuina combined therapy. The results showed significant pain improvement with Tuina alone therapy (SMD: −0.89; 95% CI −1.43 to −0.34, *P* = .01) and with Tuina combined therapy (SMD: −0.80; 95% CI −1.17 to −0.44; *P* < .001) in patients with CNLBP (Fig. 3).

#### 3.4.2. The efficacy of Tuina for physical function.

Changes in physical function in CNLBP were assessed in 13 trials, while only 6 trials utilized the Oswestry Disability Index and thus were included in the meta-analysis. Two trials investigated the effect of Tuina alone therapy on functional status,^[32,34]^ and 4 trials investigated the effect of Tuina combined therapy.^[21,31,37,40]^ Comparison groups consisted predominantly of multiple interventions (i.e., exercise, acupuncture, health education, herbal medication, conventional medication, and other combined treatments) in CNLBP.

**Figure 4.**
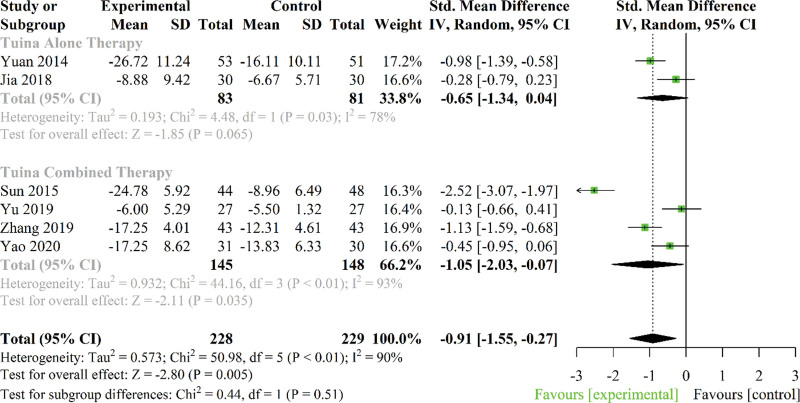
Forest plot of the effect of Tuina therapies on physical function in patients with chronic nonspecific low back pain. CI = confidence interval, SD = standard deviation.

Pooled analysis revealed significant effects of Tuina therapies on physical function improvement (SMD: −0.91; 95% CI −1.55 to −0.27; *P* = .005; *P* < .01; *I*^2^ = 90%). Subgroup analysis indicated that Tuina combined therapy significantly resulted in greater improvement in physical function compared to control group (SMD: −1.05; 95% CI −2.03 to −0.07; *P* = .035; *I*^2^ = 93%), but Tuina alone therapy did not when compared to control group (SMD: −0.65; 95% CI −1.34 to 0.04; *P* = .065; *I*^2^ = 78%) (Fig. 4).

#### 3.4.3. The efficacy of Tuina for QoL improvement.

Two RCTs utilized the 36-Item Short Form Survey to assess QoL change in CNLBP.^[36,39]^ One trial compared Tuina combined with herbal medications^[36]^ while the other trial compared Tuina combined with exercise.^[39]^

**Figure 5.**
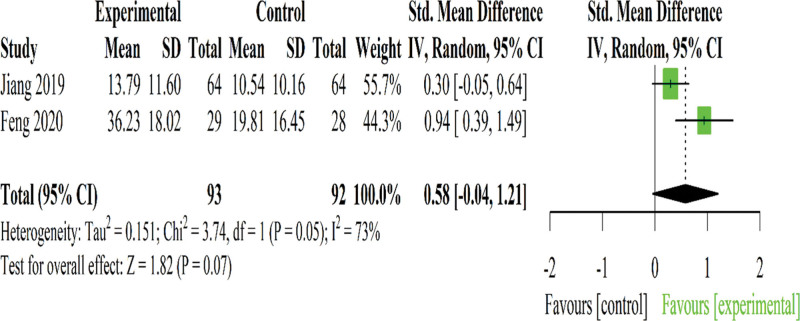
Forest plot of the effect of Tuina on quality of life in patients with chronic nonspecific low back pain. CI = confidence interval, SD = standard deviation.

Pooled analysis revealed no significant effects of Tuina combined therapies on QoL improvement (SMD: 0.58; 95% CI −0.04 to 1.21; *P* = .07; *I*^2^ = 73%) (Fig. 5).

##### 3.4.3.1. Safety and compliance.

Six trials reported adverse event information (Fig. 2).^[19,32–35,39]^ Three trials reported the occurrence of low back skin bruising, localized pain, and symptom exacerbation in the treatment groups.^[19,32,33]^ Two participants stopped the treatment due to AEs,^[19]^ while the other participants recovered spontaneously without additional medical care.^[32,33]^ Three trials descriptively reported no AEs,^[31,34,39]^ while there was no reported data of AEs recorded in the other 9 studies.^[18,21,29,30,35–38,40]^ Eight trials reported that all patients included had cooperated in completing the trial,^[18,30,31,33,35–38]^ the other seven reported that 5% (33/654) and 4% (32/736) patients had poor compliance and could not complete all of the treatment courses or were lost to follow-up^[19,21,29,32,34,39,40]^ in interventional groups and control groups, respectively.

##### 3.4.3.2. Sensitivity analysis and publication bias.

**Figure 6.**
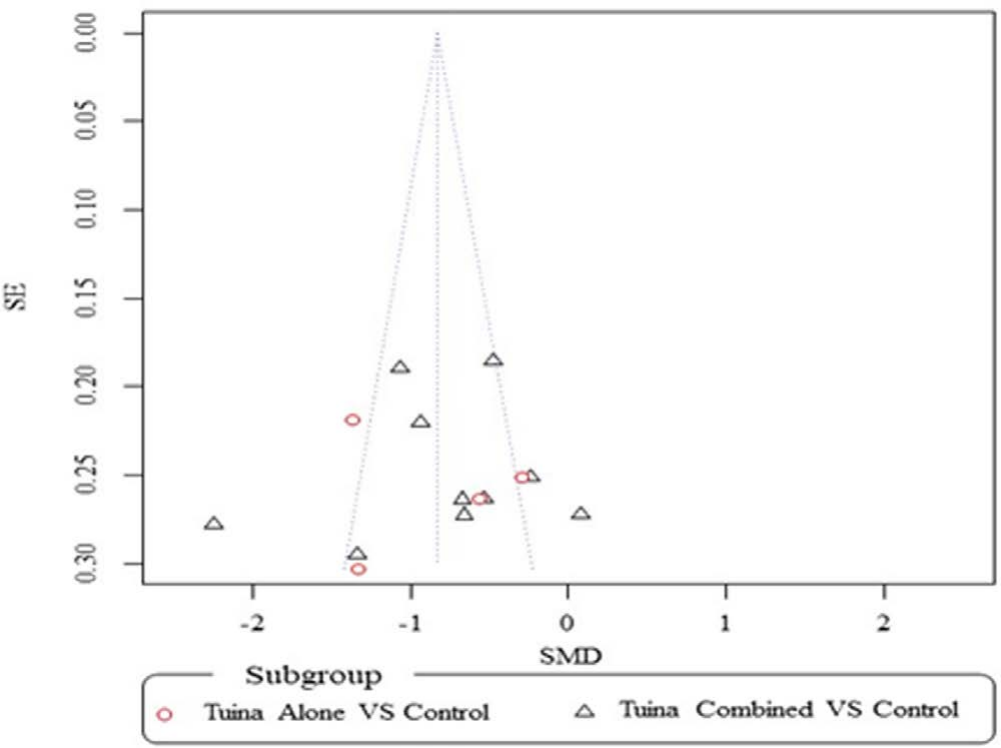
Funnel plot of comparison between Tuina vs control groups for changes in pain. SMD = standardized mean difference.

The sensitivity analysis revealed that excluding any single study would not affect the significance of our combined effect size for either outcome. The funnel plot on pain intensity showed symmetry, suggesting the risk of publication bias was unlikely (Fig. 6).

##### 3.4.3.3. GRADE quality assessment.

The GRADE quality assessment for pain, physical function, and QoL was low level (Table 3). The reasons for the evidence downgrading were due to the risk of bias from poor methodological quality (lack of allocation concealment, inability to blind, and concerns for selective reporting), high heterogeneity, and insufficient sample size. The summary of study findings and evidence quality is presented in Table 3.

**Table 3:** Tuina compared to control treatment for CNLBP.

Patient or population: CNLBP					
Setting:					
Intervention: Tuina					
Comparison: Control treatment					
Outcomes	No of participants (studies) Follow-up	Certainty of the evidence (GRADE)	Relative effect (95% CI)	Anticipated absolute effects	
				Risk with control treatment	Risk difference with Tuina
Pain intensity assessed with: VAS, NRS	1114 (14 RCTs)	⨁⨁◯◯ Low†,‡	**RR 0.99** (0.97–1.22)	957 per 1000	**10 fewer per 1000** (29 fewer to 210 more)
Disability assessed with: ODI	503 (6 RCTs)	⨁⨁◯◯ Low†,‡	**RR 0.98** (0.93–1.03)	920 per 1000	**18 fewer per 1000** (64 fewer to 28 more)
Quality of life assessed with: SF-36	188 (2 RCTs)	⨁⨁◯◯ Low†,§	**RR 1.01** (0.97–1.05)	**Study population**	
				979 per 1000	**10 more per 1000** (29 fewer to 49 more)
				**Moderate**	
				0 per 1000	**0 fewer per 1000** (0 fewer to 0 fewer)
**GRADE Working Group grades of evidence**					
**High certainty:** We are very confident that the true effect lies close to that of the estimate of the effect.					
**Moderate certainty:** We are moderately confident in the effect estimate: the true effect is likely to be close to the estimate of the effect, but there is a possibility that it is substantially different.					
**Low certainty:** Our confidence in the effect estimate is limited: the true effect may be substantially different from the estimate of the effect.					
**Very low certainty:** We have very little confidence in the effect estimate: the true effect is likely to be substantially different from the estimate of effect.					

CI = confidence interval, CNLBP = chronic nonspecific low back pain, GRADE = Grading of Recommendations, Assessment, Development and Evaluation, NRS = Numeric Rating Scale, ODI = Oswestry disability index, RCTs = Randomized controlled trials, RR = risk ratio, SF-36 = 36-Item Short Form Survey.

*The risk in the intervention group (and its 95% confidence interval) is based on the assumed risk in the comparison group and the relative effect of the intervention (and its 95% CI).

† High risk of bias due to lack of allocation concealment, blinding and selective reporting.

‡ Significant heterogeneity.

§ Few events.

## 4. Discussion

To our knowledge, this review is the first systematic review and meta-analysis assessing the efficacy and safety of Tuina in CNLBP. The authors hypothesized that Tuina was an effective and safe intervention for CNLBP, and the extracted evidence supported our hypothesis. The included studies found that Tuina could mitigate pain and physical disability, but not improve QoL in CNLBP. Our findings are consistent with the findings of a recent systematic review and meta-analysis performed by Kong et al,^[17]^ that assessed twenty RCTs regarding the effectiveness of Tuina-focused integrative Chinese medical therapies in patients with LBP (as compared to CNLBP in the present review). They concluded that Tuina-focused integrative Chinese medical therapies might be effective in LBP.

When considering the safety of Tuina treatment, AEs were reported in 20% of the included studies and mainly were localized skin bruising, pain, and symptom aggravation. All AEs resolved spontaneously without additional medical intervention and no severe AEs occurred. However, a thorough literature review did identify reports of headache, nausea, vomiting, broken skin, bleeding, syncope, hematoma, and fracture associated with Tuina, albeit very rarely.^[41–43]^ Therefore, Tuina seems to be a relatively safe intervention, making it easy to be accepted and adhered to with high compliance.

Our review complied with the Cochrane Handbook for Systematic Reviews of Intervention version 6.2. The formulation of our review highlighted some of the issues surrounding Tuina and CNLBP research in general. We excluded several studies comparing different Tuina techniques, trials not focusing on Tuina or including Tuina in control groups, or trials that did not include CNLBP. These factors prevented us from including these studies in our analysis, since it would have been near impossible to decipher the sole impact of Tuina on CNLBP due to various potential confounders.

Although a variety of studies demonstrate the effect of Tuina on pain and physical function in CNLBP, the specific underlying therapeutic mechanism of action of Tuina remains unclear. The mechanism of Tuina could be related to relaxing muscles and tendons, improving local blood circulation, regulating spinal balance or alignment, or decreasing edema and inflammation.^[44–46]^ Some of the included trials reported on factors such as electromyographic correlates,^[35]^ β-EP, IL-6, TNF-α,^[39]^ and the median frequency of surface electromyography^[40]^; these factors may contribute to the underlying therapeutic mechanism of Tuina. Additional well-designed studies are needed to further explore the potential therapeutic mechanisms of Tuina.

It should be noted that the level of evidence of this meta-analysis was determined to be low, indicating that these findings should be interpreted with caution. The high heterogeneity between studies cannot be ignored, especially regarding Tuina interventions utilizing various techniques, duration, and frequency of treatments. In addition, although all included studies were RCTs, they were rated as “high” or “unclear” risk of bias. Due to the methodological limitations (lack of participant random allocation, lack of blinding and outcome reporting bias), the quality of the outcome evidence was downgraded to “low certainty,” which might impact the accuracy and reliability of the evidence.

Some limitations should be also noted when interpreting the study results. This is the first tentatively comprehensive review that solely collected and appraised available literature relating to clinical studies investigating Tuina for CNLBP. However, only English and Chinese databases were searched, so additional studies could have been overlooked. All the trials were performed in China, and most of them were written in Chinese, which needed to be translated into English for further analysis. Errors in translation and subtext are possible. Furthermore, given that all trials took place in China, we are limited in our generalizability and applicability of these findings. Future studies should be well-designed, standardized (in terms of Tuina therapy, duration, and frequency), methodologically rigorous, and include a racially diverse cohort of participants with CNLBP.

## 5. Conclusions

In conclusion, Tuina might have a positive effect in reducing pain and physical disability in people with CNLBP, but not on QoL. Mild AEs were reported sparsely and resolved spontaneously without special medical interventions. However, the overall certainty of the study results was limited due to poor methodological quality, high heterogeneity, and insufficient sample size of the included trials. Well-designed multicenter RCTs are needed to confirm our findings in the future.

## Acknowledgments

The authors thank Prof Tay Boon Keng, the previous Chairman, Medical Board, International, SingHealth, for his initial support of Tuina treatment for low back pain. The authors also thank the experienced librarian Larry J. Prokop, M.L.S., Mayo Clinic Rochester Campus for the literature research of this review.

## Author contributions

**Data curation:** Juan Yang, Xuan Zhou, Qingyu Ma.

**Formal analysis:** Juan Yang, Xuan Zhou, Qingyu Ma.

**Investigation:** Celia Ia Choo Tan, Jiaxu Chen, Brent A. Bauer.

**Methodology:** Alexander Do, Jeffrey S. Brault, Mark A. Jensen, Longbin Shen, Yu Guo, Shujie Tang.

**Project administration:** Brent A. Bauer.

**Software:** Kejie He, Zhuoming Chen.

**Supervision:** Celia Ia Choo Tan, Jiaxu Chen, Brent A. Bauer.

**Writing – original draft:** Juan Yang.

**Writing – review & editing:** Jeffrey T. Woods, Arya B. Mohabbat, Kyung-Min Shin, Canghuan Zhao, Kwok Chee Philip Cheong, Yong Tang.
